# Wrangling Shape-Shifting Morpheeins to Tackle Disease and Approach Drug Discovery

**DOI:** 10.3389/fmolb.2020.582966

**Published:** 2020-11-27

**Authors:** Eileen K. Jaffe

**Affiliations:** Fox Chase Cancer Center, Philadelphia, PA, United States

**Keywords:** protein structure dynamics, allostery, morpheein, fifth level of protein structure, drug discovery

## Abstract

Homo-multimeric proteins that can come apart, change shape, and reassemble differently with functional consequences have been called morpheeins and/or transformers; these provide a largely unexplored context for understanding disease and developing allosteric therapeutics. This article describes such proteins within the context of protein structure dynamics, provides one detailed example related to an inborn error of metabolism and potential herbicide development, and describes the context for applying these ideas for understanding disease and designing bioactive molecules, such as therapeutics.

## Introduction

A great number of medically relevant proteins are homo-multimers, some of which exist as an equilibrium of alternate assemblies that are both non-additive and functionally distinct. The phenomenon wherein protein homo-multimers can come apart, change shape while dissociated, and reassemble into an architecturally and functionally different assembly has been called the morpheein model of protein allostery (Jaffe, 2005; see Morpheein in Wikipedia^1^). A key to this protein structure dynamic is that the required conformational change is *spatially forbidden* within the context of either assembly. Proteins with this capacity can be called morpheeins and the alternate assemblies can be called morpheein forms. The dynamic process of dissociation and association makes this mode of allostery distinct from the classic Monod-Wyman-Changeux and Koshland-Nemethy-Filmer models; it provides a conceptually distinct approach to understanding normal protein function, disease-associated protein dysfunction, drug action, and approaches to drug design. This article describes the morpheein model for allosteric regulation, provides a disease relevant example in the protein porphobilinogen synthase, and considers current and future research intended to capitalize on targeting quaternary structure shape shifting in many different proteins as a way to understand disease and develop therapies. Because there are so few well characterized examples, even the most comprehensive treatments of allosteric drug discovery do not address proteins that are established to sample a dynamic equilibrium of assemblies comprised of alternate protomer conformations whose interconversion is forbidden within the assemblies (Lu et al., 2019a,b).

## Morpheeins Within the Context of Protein Structure Dynamics

The existence of morpheeins is one of many protein structure dynamic phenomena that falls outside the classic one sequence – one structure – one function paradigm. Consequently, such discoveries have been surprising (e.g., Breinig et al., 2003; Kashlan and Cooperman, 2003; Bornholdt et al., 2013), often serendipitous, and have been accompanied by the introduction of alternate related nomenclature such as transformers and metamorphic proteins (e.g., Murzin, 2008; Lopez-Pelegrin et al., 2014; Wasserman and Saphire, 2016; Dishman and Volkman, 2018). These refer to a continuum of quaternary structure dynamics which expand our view of protein structure beyond the level of primary, secondary, tertiary and quaternary. In the study of ribonucleotide reductase as a drug target, the investigator Aye has referred to going beyond quaternary structure as “breaking the fourth wall”; in the study of Ebola virology, the investigator Ollmann-Saphire has termed it the “fifth level of protein structure” (Wasserman and Saphire, 2016; Long et al., 2019). Herein, we use the term “fifth level of protein structure” to refer to equilibria of alternate assemblies comprised of alternate protomer conformations. This builds on the established concept that protein function is a consequence of an equilibrium of protein structures (Parisi et al., 2015). Both Aye’s and Ollmann-Saphire’s treatments highlight that normal protein structure dynamics can include architecturally distinct assemblies with alternate functions that are comprised of different protomer conformations. These assemblies exist as equilibria in the absence of chemical modification. The populations (e.g., mole fraction) of alternate morpheein forms responds to environmental factors (e.g., ionic strength, pH) and most significantly to ligand binding. These factors may govern the predominance of alternate morpheein forms in different cellular locations. Single amino acid substitutions that alter the mole fractions of alternate morpheein forms can cause disease (e.g., Jaffe and Stith, 2007). The morpheein model of protein allostery is a dissociative allosteric model most closely related to the equilibrium models of Nussinov (e.g., Kar et al., 2010) and Hilser (e.g., Motlagh et al., 2014), with the added dimension of quaternary structure. In the prototype morpheein described below, porphobilinogen synthase, the alternate functions are high activity (on) vs. low activity (off) (Jaffe and Stith, 2007; Jaffe and Lawrence, 2014; Jaffe, 2016, 2020). In the Ebola virus VP40 protein, the alternate functions are entirely separate activities, each one of which is essential for the viral life cycle (Bornholdt et al., 2013). Proteins that can moonlight (carry out unrelated functions, like VP40) were first discovered in the 1980’s (e.g., Gurney et al., 1986), and often arose from cloning the gene responsible for a biological function only to discover that the cloned protein sequence was already known to have a different function. A fascinating example is the protein originally identified as the glycolytic enzyme glyceraldehyde-3-phosphate dehydrogenase, which now has many documented functions, many of which can be targeted for drug discovery (e.g., Kopeckova et al., 2020; Lazarev et al., 2020). The known moonlighting proteins have recently been assembled by the investigator Jeffery into a MoonProt^®^ database, which currently has ∼400 listings (Mani et al., 2015; Chen et al., 2018). In most instances it remains to be determined if alternate moonlighting functions are associated with alterations at the fifth level of protein structure. A related fifth level phenomenon is the reversible filamentation of some enzymes, recently reviewed by Horton (Park and Horton, 2019, 2020). Outstanding questions for many filament-forming proteins is whether they are morpheeins (with alternate protomer conformations), moonlighting proteins (with more than one function), or both. Two related enzymes, CTP synthase and IMP dehydrogenase are exemplars of this unknown. Each, separately and together, undergo changes in multimerization or filament formation in response to the state of the cell, but functional distinctions among these assemblies are yet unknown (Simonet et al., 2020).

**FIGURE 1:**
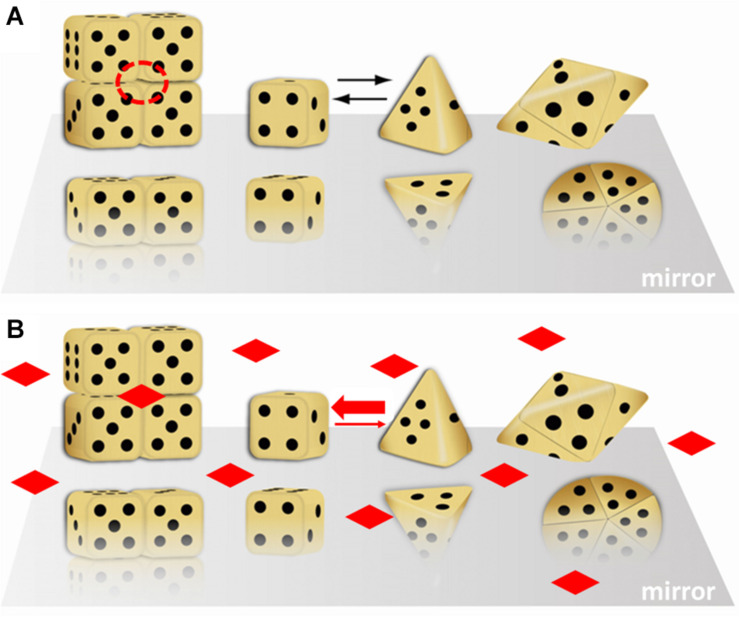
A dice based illustration of the morpheein phenomenon. That the alternate assemblies have different functions makes this a dissociative model for protein allostery. In this illustration, the stoichiometry of the alternate assemblies is different; this is just an example, not a prerequisite. **(A)** Cubic and pyramidal dice are used as symbolic (not structural) representations of alternate conformations of a protomer that can self-assemble through association of two complementary surfaces. In this case, the two surfaces are represented by the die face with one and with four dots. The tetramer resembles a stack of boxes; the pentamer resembles a flying saucer. The red dashed circle is a multimer-specific surface cavity that can serve as a ligand binding site. Note that the orientation of the protomer is not retained in the illustration of the multimer. **(B)** The diamond shaped ligands can bind to the multimer-specific binding site and draw the equilibrium toward the assembly of cubic dice, thus dictating protein function. This figure was adapted (with permission) from an image first printed as the journal cover associated with (Lawrence et al., 2008).

Figure 1 illustrates the morpheein phenomenon using differently shaped dice to represent different conformations of the protomer. Monod first used dice assemblies to illustrate quaternary structures (Monod, 1965). Figure 1 shows equilibration between two alternate conformations of the protomer, where one is represented by a cubic die and the other is represented by a pyramidal die. Although not obvious from the representative shapes, *the interconversion of these conformations does not require any substantial changes in the protein fold at the level of secondary or tertiary structure. The interconversion is spontaneous; it does not require any external input of energy*. It may involve small regions of order – disorder transition. For example, interconversion between the protomer conformations represented by the cube and pyramid could be a hinge motion between two folded domains of each protomer that allows the die face with five dots to associate with the die face with six dots, burying these surfaces. Hinge motions allow protomer shape change without requiring a change in protein fold. In Figure 1, the higher order multimers form by association of the die face with one dot to the die face with four dots. The cubic die forms a symmetric tetramer; the pyramidal die forms a symmetric pentamer. It is easy to imagine how these two assemblies, though made up of chemically identical components, will interact with different cellular partners and potentially have different functions. Note that the surface of the tetramer contains a multimer-specific surface cavity that can serve as a ligand binding site (dashed circle in Figure 1A). The ligand could be a natural allosteric effector molecule, a drug, or another cellular entity (protein, nucleic acid, lipid, membrane surface). The pentamer does not have this same surface cavity and will not interact with the same ligand. In Figure 1B, addition of the imagined ligand causes stabilization of the tetramer, which will draw the structural equilibrium toward the tetramer and alter the protein’s function to that of the tetramer. All of this happens in the absence of post-translational modifications or any other covalent changes to the protein. We note, however, that changes in protein sequence, post-translational modification, or the presence of purification tags can shift the position of the equilibrium (mole fraction of alternate morpheein forms) and enhance or inhibit allosteric ligand binding.

## The Prototype Morpheein – Porphobilinogen Synthase (PBGS)

### PBGS Provides a Physiologic Relevance to the Morpheein Model of Allostery

**FIGURE 2:**
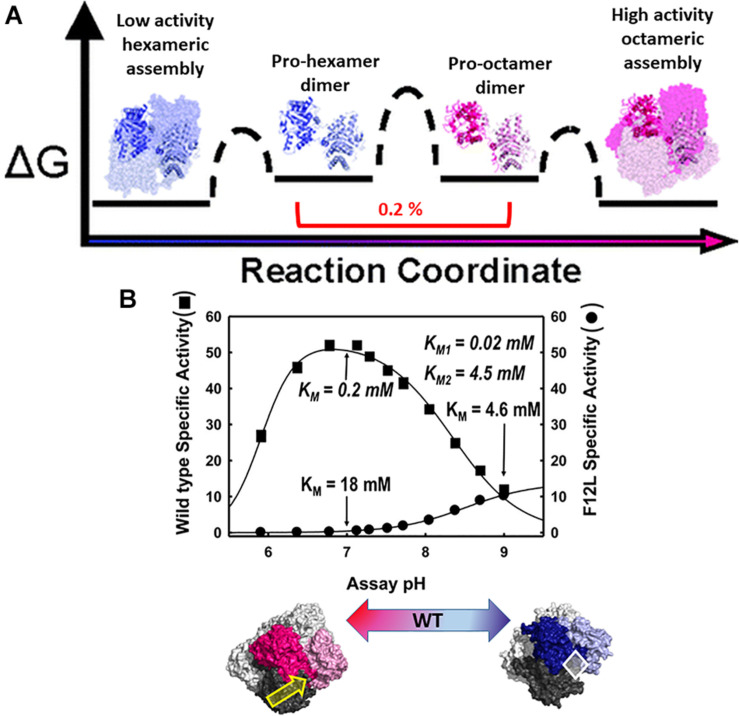
The equilibrium of alternate morpheein forms of PBGS. In all panels, octamer (or pro-octamer dimer) components are in shades of pink; hexamer (or pro-hexamer dimer) components are in shades of blue. **(A)** The reaction coordinate diagram for the interconversion of human PBGS morpheein forms [adapted with permission from Selwood et al. (2008), copyright 2008, American Chemical Society]. In this illustration, one dimer is shown in shaded ribbons (darker/lighter) while the other dimers are in space filling with the same coloring. Note the low mole fraction of the essential dimeric intermediates (Selwood et al., 2008). **(B)** The pH rate profile (10 mM substrate, Bis-tris propane buffer) for WT human PBGS and the disease-associated F12L variant, which strongly favors the hexameric assembly [images adapted from Breinig et al. (2003)]. K_M_ and V_MAX_ determinations varied substrate from 10 μM to 10 mM at pH 7 and pH 9. Below illustrates that the position of the quaternary structure equilibrium is a function of pH; at neutral pH the WT protein is predominantly octamer while at pH 9, it is predominantly hexamer. The pH dependent transition from octamer to hexamer for WT human PBGS accounts for the basic arm of the pH rate profile (Selwood et al., 2008). A key component of this pH dependence is the protonation of an arginine residue, which sits at a multimer specific interface between the N-terminal arm of the dark pink subunit and the αβ-barrel of the dark gray subunit, as indicated by the yellow arrow (Tang et al., 2006). This arginine is spatially equivalent to an allosteric magnesium ion found in PBGS from species that are neither metazoan nor fungal. The octamer-specific interface stabilizes a closed active site lid. The white diamond indicates the hexamer-specific surface cavity that was successfully targeted for identification of species-specific (plant) PBGS inhibitors (Lawrence et al., 2008).

The physiologic relevance of the morpheein model of allostery was first realized for the protein porphobilinogen synthase (PBGS), whose quaternary structure dynamic is illustrated in Figure 2A (Breinig et al., 2003; Selwood et al., 2008). Here I paint a broad picture of PBGS, highlighting key aspects of its fifth level of protein structure and refer the reader to recent reviews for more details (Jaffe and Lawrence, 2014; Jaffe, 2016). In the PBGS example, optimal enzyme activity requires controlled access to the enzyme active site, which is gated by the opening and closing of an active site lid. Each protomer has a complete active site, but securely closing the active site lid depends upon a network of molecular interactions that can only be achieved in the octamer (Jaffe, 2016). In the PBGS example, the alternate assemblies have different multimer-specific surface cavities that can be used for the development of bioactive molecules. The alternate assemblies also have different size, shape, and surface charge, which allows them to be separated by biochemical and biophysical methods such as native PAGE and ion exchange chromatography (Breinig et al., 2003).

The PBGS catalyzed reaction is essential for all organisms that rely on methanogenesis, photosynthesis, and/or respiration, thus covering every branch of cellular life. With all this evolutionary time to adapt to the organism’s needs and to function in different cellular environments (e.g., cytoplasm, chloroplast, apicoplast), factors governing the fifth level of PBGS protein structure are not evolutionarily conserved. Additionally, the amino acid composition of the targeted surface cavities is not conserved, unlike active site residues. This makes the allosteric regulation of PBGS a potential target for the development of antimicrobials and herbicides. In some species (e.g., plants, bacteria, archaea) the equilibrium position depends upon an allosteric magnesium binding at an interface only present in the octamer (see yellow arrow in Figure 2B). In PBGS from metazoa and fungi, which lack the magnesium binding site, in its place is the guanidinium group of an arginine residue. In the human PBGS variant where a leucine is substituted for Phe12 (F12L), from which we obtained the crystal structure of the hexameric assembly (Breinig et al., 2003), this conserved arginine does not contact the neighboring N-terminal arm. In an apicoplast, PBGS has evolved to contain a C-terminal extension that prevents the hinge motion necessary to convert pro-octamer dimer to pro-hexamer dimer; this hinge motion is illustrated in Figure 2A. Thus, in apicoplast PBGS the equilibrium components are limited to octamer and pro-octamer dimer (Jaffe et al., 2011). This phylogenetic variation demonstrates that evolution of the fifth level of protein structure provides another opportunity for the adaptation of protein functional control.

### Control of PBGS Morpheein Forms by pH and Ligand Binding

The human PBGS pH rate profile (Figure 2B) helped reveal a pH dependence to the quaternary structure equilibrium of wild type human PBGS (see the bottom panel of Figure 2B; Selwood et al., 2008). The crystal structures of the PBGS octamer (e.g., Jaffe, 2004; Jaffe et al., 2011; Mills-Davies et al., 2017) show that a non-reacting moiety of the K_M_-determining substrate interacts with the active site lid, essentially closing the lid and allowing deprotonation of an active site lysine required for essential Schiff base formation. With an open active site, this deprotonation requires a high solvent pH, as is seen in the pH rate profile of the constitutively hexameric F12L variant. The crystal structures of the protomers in the human PBGS octamer vs. hexamer superpose remarkably well, but differ in two key ways. First is the backbone hinge between the αβ-barrel and the N-terminal arm domains; this dictates assembly to octamer vs. hexamer. Second is the presence of an ordered active site lid, which is only present in one hemisphere of the octamer. Both structures contain active site ligands in a half-of-the-sites stoichiometry (one hemisphere) (PDB: 1E51, 1PV8). Only the ligand-containing octamer active sites contains atoms that derive from the K_M_-determining substrate, which are securing a closed conformation of the active site lid through a network of bonds between the substrate’s carboxyl group and basic residues on the lid.

**FIGURE 3:**
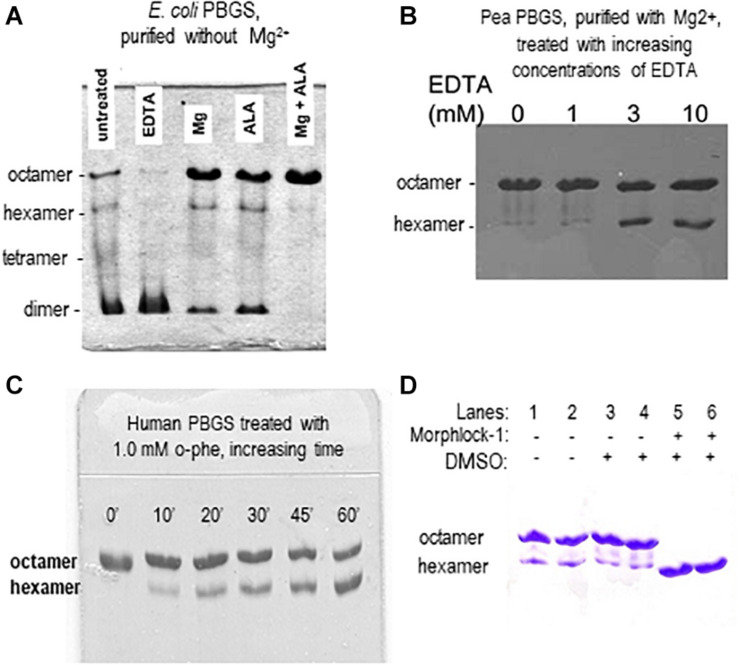
Native PAGE illustrates how allosteric ligands control the distribution of PBGS morpheein forms. **(A)** The distribution of morpheein forms of *E. coli* PBGS responds to magnesium and substrate (ALA), both of which stabilize the octamer [image adapted with permission from Jaffe et al. (1995), copyright 1995 American Chemical Society]. In this example the protein was purified with zinc, but not magnesium (Jaffe et al., 1995). The *E. coli* PBGS octamer and the position of the allosteric magnesium are established by crystal structure (Kervinen et al., 2001). Hexamer, tetramer, and dimer positions are modeled on the assumption that mobility of all assemblies is governed by the same charge/mass ratio. **(B)** The distribution of morpheein forms of plant PBGS (*Pisum sativum*) responds to the addition of EDTA, which strips the magnesium, destabilizing the octamer and favoring accumulation of hexamer [image adapted from Breinig et al. (2003)]. In this example the protein was purified in the presence of magnesium (Kervinen et al., 2000; Breinig et al., 2003) and multimer size was determined by analytical ultracentrifugation (Kokona et al., 2008). **(C)** Binding the K_M_ determining substrate of human PBGS relies upon an active site zinc ion. Removing the zinc with 1,10-phenanthroline destabilizes the octamer [image adapted with permission from Jaffe (2016) copyright 2016 American Chemical Society]. **(D)** A ligand discovered through computational docking to a hexamer-specific binding site on plant PBGS stabilizes the hexamer [image adapted with permission from Lawrence et al. (2008)]. This ligand inhibits the plant enzyme, but does not affect the activity of the human enzyme [not shown, Lawrence et al. (2008)].

Although the PBGS quaternary structure equilibrium is controlled by different factors in different branches of life, there are unifying characteristics in the pH rate profiles of alternate PBGS morpheein forms. Mammalian PBGS at neutral pH (see Figure 2B, bottom), and plant/bacterial PBGS with magnesium present are predominantly octameric and are documented to have K_M_ values in the range of ∼150 μM (Mitchell and Jaffe, 1992; Jaffe et al., 1995; Breinig et al., 2003), which is the range of the cellular substrate concentration. The isolated hexameric F12L variant (see Figure 2B), and the bacterial *Escherichia coli* PBGS without magnesium exhibit K_M_ values (at neutral pH) that are well above the physiological substrate concentration, in the range of 5–20 mM. Addition of magnesium to a magnesium-free *E. coli* PBGS sample has been shown to shift the quaternary structure equilibrium to octamer (Figure 3A) and reduce the K_M_ to that characteristic of octamer (Mitchell and Jaffe, 1993; Jaffe et al., 1995). Similarly, treatment of a plant PBGS with EDTA causes a shift from octamer to hexamer (Figure 3B; Breinig et al., 2003). For human PBGS, this same phenomenon can be demonstrated by removal of a catalytic zinc ion that is essential for binding the K_M_-determining substrate (Figure 3C; Jaffe, 2016). The high K_M_ values for PBGS that are not octameric derives from an inability to secure the closed active site lid, leaving the K_M_-determining substrate loosely bound. What is striking about the pH rate profile of WT human PBGS is the shift in K_M_ values between pH 7 and pH 9, where the kinetic parameters resemble that of hexameric F12L. In fact, WT human PBGS at pH 9 exhibits a double hyperbolic kinetic behavior. At low substrate concentration, the observed reaction rate is dominated by the low K_M_ (high V_MAX_) octameric component, which is at a relatively low mole fraction. As the substrate concentration starts to approach the K_M_ of the hexamer, the observed enzyme catalyzed reaction rate become dominated by the high K_M_ (low V_MAX_) hexameric component (which is at a high mole fraction). This double hyperbolic kinetic phenomenon, caused by a mixture of species with different kinetic constants follows a classic treatment of isozymes. The alternate morpheein forms of PBGS are not isozymes. Rather, they are a slowly exchanging mixture octamer and hexamer.

### An Inborn Error of Metabolism Is Linked to Perturbation of an Equilibrium of Morpheein Forms

**FIGURE 4:**
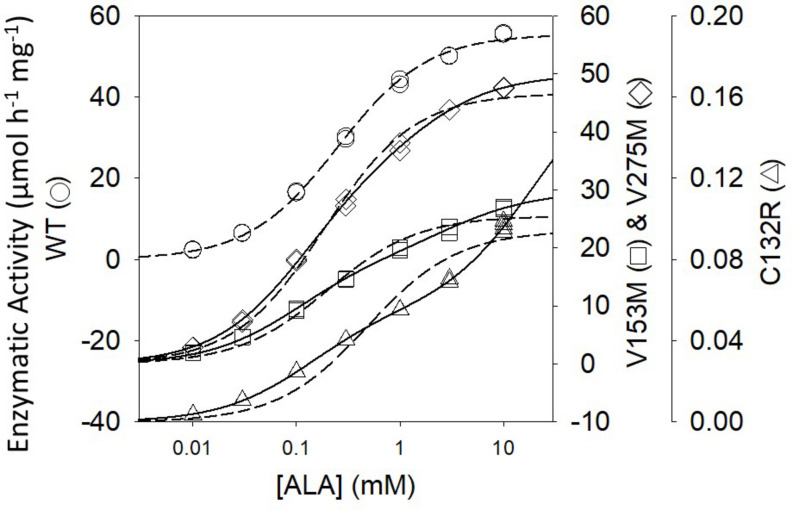
The activity vs. substrate relationship for disease-associated human PBGS proteins indicate the presence of two morpheein forms at neutral pH. The dashed lines are fitted to the hyperbolic Michaelis–Menten equations, which is an excellent fit for the WT protein. The solid lines are double hyperbolic fits (the sum of two species with different K_M_ and V_MAX_ values) [image adapted with permission from Jaffe and Stith (2007)]. The kinetic behavior of each variant shows evidence for a mixture of low K_M_ and high K_M_ morpheein forms, corresponding to octamer and hexamer (Jaffe and Stith, 2007). The wild type protein shows this double hyperbolic behavior at pH 9 (see Figure 2B).

ALAD porphyria is a rare inborn error of metabolism caused by dysfunctional PBGS and inherited as a recessive disease (Maruno et al., 2001). ALAD, an abbreviation for amino-levulinic acid dehydratase, is an alternate name for PBGS; it remains in clinical use. There are eight known disease-associated variants and all patients are compound heterozygotes. Only one disease-associated variant alters an amino acid at the enzyme active site. When heterologously expressed and purified from *E. coli*, the disease-associated human PBGS variants all show an increased propensity to populate the hexameric assembly at neutral pH (Jaffe and Stith, 2007). This can be illustrated by the appearance of the double hyperbolic kinetic phenomenon at neutral pH (Figure 4), which is indicative of a mixture of octamer and hexamer. It is also seen by native PAGE and ion exchange chromatography, both of which separate the octamer from the hexamer, as confirmed by the established crystal structures of the wild-type and the constitutively hexameric F12L variant (Breinig et al., 2003).

We have posited that the disequilibrium of alternate assemblies contributes to the complex phylogenetic patterns seen in the most common inborn error of amino acid metabolism, phenylketonuria, which is caused by dysfunctional phenylalanine hydroxylase (Jaffe et al., 2013; Jaffe, 2017). Although it is now established that phenylalanine hydroxylase can equilibrate between architecturally distinct high activity and low activity tetrameric assemblies, it is not yet established whether tetramer dissociation is a required component their interconversion (Jaffe, 2017).

## We Could Easily Have Missed the Fifth Level of Protein Structure When Studying PBGS

The four levels of protein structure are introduced in every biochemistry text. Additionally, the relationship between sequence, structure, and function is the foundation of broad applications of bioinformatics, which drive much biomedical research. For the first 20 years that we studied PBGS, we were not looking for the fifth level of protein structure. All of our data prior to ∼2003 was interpreted within the context that PBGS has one fixed quaternary architecture, which is an octamer (e.g., Wu et al., 1974), in possible equilibrium with tetramers or dimers comprised of the same protomer conformation. For example, our published model of the protein concentration dependence of a plant PBGS included only dimer, tetramer, and octamer (Kervinen et al., 2000). A few publications had suggested that PBGS is a hexamer (e.g., Stolz and Dornemann, 1996). But, we believed that one of these interpretations must be incorrect.

The first PBGS crystal structure, published in 1997, showed the architecture of the octamer, establishing precedent, as crystal structures often do (Erskine et al., 1997). As described above, the third decade of our work with PBGS revealed the protein as the prototype morpheein. We would not have seen this but for a confluence factors. (1) With a focus on the evolution of metal ion usage, we had correlated sequence variations with *in vitro* behaviors looking at PBGS from mammals, bacteria, and plants (e.g., Jaffe, 2003). By the time we fully evaluated this relationship, the first crystal structure of a bacterial PBGS showed the location of the allosteric magnesium binding site (Frankenberg et al., 1999). It is present in the octamer and absent in the hexamer. (2) We had not used purification tags, which allowed ion exchange chromatography to reveal the separation of PBGS morpheein forms during purification. It was a surprise when the disease-associated PBGS variant F12L had a very different mobility on an ion exchange column relative to the charge-equivalent WT protein (Breinig et al., 2003). (3) Had we not chosen to study the F12L variant, we would not have had the stable hexamer as a reagent for comparative analysis. For decades we had been discarding the small amount of low activity hexameric mammalian PBGS that is present at neutral pH and separated during our purification of wild type proteins. When studying a human PBGS variant from which the catalytic zinc binding site had been removed, we consistently saw two peaks on an ion exchange column, but could not discriminate these by circular dichroism or enzyme kinetics. By kinetic criteria they were the same, because addition of substrate converted the hexamer to the octamer during the long time-course assays for these very low activity variants. Substrate stabilization of the octameric assembly can be illustrated using 2-dimensional native PAGE during which the gel is incubated in assay mixture between the two dimensions (e.g., Jaffe et al., 1995; Lawrence et al., 2008; see also Figure 3A). (4) Had we not obtained the crystal structure of F12L, we would not have seen the structure of the hexamer. From this, and knowledge of the location of the allosteric magnesium from prior crystal structures, we realized the octamer ↔ hexamer equilibrium as the basis for allosteric regulation of PBGS. Our extensive work on *E. coli* PBGS (including the 1995 documentation of the native PAGE phenomenon shown in Figure 3A) could now be reinterpreted within the context of this new allosteric model. Having previously interpreted our data in terms of a classic primary, secondary, tertiary, and quaternary structure paradigm, we had overlooked hints that the PBGS octamer was part of an equilibrium of differently sized morpheein forms. For example, a ^13^C NMR study of labeled substrate bound to *E. coli* PBGS (in the absence of magnesium) had unexpectedly yielded NMR line widths significantly narrower relative to an octameric mammalian PBGS (Jaffe and Markham, 1988; Mitchell and Jaffe, 1993), suggesting a faster rotational correlation time indicative of a smaller multimeric size.

## Finding Proteins That Experience the Fifth Level of Protein Structure

Since the fifth level of protein structure provides an additional way to manipulate protein function, it also provides an additional approach to allosteric drug discovery. Consequently, there is value in identifying proteins that behave as morpheeins. This goal remains difficult. The initial discovery of the morpheein character of PBGS was serendipitously based on *in vitro* protein behavior coupled with X-ray crystal structures showing architecturally different assemblies comprised of different protomer conformations, but not different folds. This discovery was not based on a bioinformatics approach. The PBGS example presented itself because there happened to be a naturally occurring variant, F12L, that sufficiently stabilized the hexameric assembly to obtain its crystal structure. Although the wild-type human PBGS was later realized to readily equilibrate between octamer and hexamer in a pH-dependent fashion (Figure 2), to date all conditions that have yielded diffraction quality crystals of the wild-type protein favored crystals comprised of octamer. Serendipity favored our studying F12L; we could not have predicted the effect of this mutation. Well-established computational approaches (e.g., the program FoldX; Schymkowitz et al., 2005) which analyze the overall thermodynamic effect of amino acid substitutions will fail to predict substitutions that shift the position of an equilibrium of morpheein forms. A similar story applies to the discovery of alternate morpheein forms and alternate functions of the Ebola virus VP40 protein (Bornholdt et al., 2013).

Our initial approach to identifying proteins that use the fifth level of protein structure was to manually search the literature for proteins that have one or more characteristics that we had documented for PBGSs. This approach was especially challenging as most of these characteristics were not contained in the searchable abstract or keywords and much of the older literature was not yet available as text-searchable PDF documents. Some of the characteristics we focused on are listed in Table 1; the characteristics included in Table 1 are each consistent with an equilibrium of alternate assemblies, but none are strictly diagnostic. Each of these behaviors can be otherwise attributed. Trevor Selwood’s herculean efforts generated boxes of reprints and a list of putative morpheeins (Selwood and Jaffe, 2012). This list, originally posted on Wikipedia (Morpheein^1^), to date has not been actively edited by the community. Failure to gain research support for using the literature to identify proteins that function as morpheeins turned this to a back-burner approach. Yet, the relatively small number of established morpheeins precludes designing a bioinformatics method for their identification. Jeffrey has described the same dilemma for the development of a bioinformatics approach to identifying moonlighting proteins (Mani et al., 2015).

**TABLE 1:** Protein behaviors that might indicate an equilibrium of morpheein forms.

**Characteristic**	**Comments**
SDS-pure protein separating into alternate bands on native PAGE can indicate multimers of alternate stoichiometry (or conformation).	Native PAGE can be routinely incorporated into the final stages of protein characterization.
SDS-pure protein that separates into alternate forms using ion exchange chromatography	This has been used to monitor the interconversion of alternate assemblies as a function of ligand.
The enzyme-kinetic phenomenon known as hysteresis.	This can be observed if the transition from a low activity form to a high activity form occurs during the assay [as we have shown for the R240A variant of PBGS (Tang et al., 2006].
Double hyperbolic kinetics (the sum of two hyperbola with different kinetic constants).	This can indicate alternate morpheein forms with different kinetic constants. Seeing this may require using a broad range of substrate concentration (see Figure 4).
X-ray crystal structures of multi-domain proteins that cannot be superposed without clashes. This observation is often dismissed as an artifact of crystal packing.	Many multi-domain proteins do not produce diffraction quality crystals. A common approach is to truncate one or more domains. If overlaying the common elements of such structures causes domains to clash, this could be a sign of alternate assemblies. This was observed for alternate truncated constructs of HIV integrase (Andrake and Skalka, 2015).
Protein concentration dependent specific activity can indicate alternate activities associated with different multimeric stoichiometries.	This is seen for all PBGS that use only magnesium [e.g., (Petrovich et al., 1996; Kervinen et al., 2000; Shanmugam et al., 2010)].
Evidence for soluble protein multimers that dissociate along a hydrophilic protein-protein interface.	This is a difficult characteristic to search for as many crystal structure files are at insufficient resolution to position water molecules. PBGS crystal structures contain phylogenetically conserved water molecules at the subunit-subunit interfaces that dissociate upon formation of the dimeric morpheein forms (Selwood et al., 2008).

Nevertheless, a key question remains as to how we can use what we have learned from PBGS to harness the fifth level of protein structure for drug discovery. In the PBGS example, the difference between the protomer that forms a hexamer and the protomer that forms an octamer is a hinge between two domains, without significantly altering the fold of these domains. This is also the case for alternate assemblies of the HIV integrase protein, where a hinge motion dictates formation of a core-core-dimer vs. a reaching dimer; and where it has been pointed out that small molecule stabilization of one or the other dimer, to prevent their interconversion, could yield a therapeutic (Bojja et al., 2013). For integrase, this promise has not yet materialized. In the case of VP40, alternate multimerization architectures also appear to arise from hinge motions between two domains, without significant refolding (Bornholdt et al., 2013). In the VP40 example, ligand association (RNA or membrane) dramatically stabilize one assembly relative to another allowing the protein to fulfill different essential function in the viral life cycle. Although not yet realized, drugs that prevent the interconversion of such assemblies could form the basis of a therapeutic. Research to facilitate this approach is ongoing (e.g., Buzon et al., 2020). In other cases wherein allosteric drugs could be imagined to work by stabilizing one of alternate assemblies (such as ribonucleotide reductase) the detailed molecular structures of alternate assemblies are only beginning to be revealed. Some of these are described in two recent reviews on the rich oligomeric and functional repertoire of both mammalian and bacterial ribonucleotide reductase enzymes (Thomas et al., 2019; Long et al., 2020).

## Identifying Allosteric Regulators (E.G., Therapeutics)

Using PBGS as an example, we have demonstrated how an equilibrium of morpheein forms can be manipulated in ways related to drugs. Realistically, ALAD porphyria is such a rare disorder that finding an octamer-stabilizing allosteric effector is, at best, an academic exercise. However, stabilization of a PBGS hexamer could form the basis for an antimicrobial or herbicide. To test this hypothesis, we targeted a hexamer-specific surface cavity on the model of a plant PBGS hexamer (*in silico* docking/*in vitro* testing) and found a hexamer-stabilizing inhibitor that did not affect human PBGS (Lawrence et al., 2008). Figure 3D illustrates the hexamer-stabilizing effect of the discovered compound (named morphlock-1). Unlike the enzyme active site, the multimer specific surface cavities is not phylogenetically conserved. We also used native PAGE to screen libraries of approved drugs as well as environmental contaminants (Lawrence et al., 2011, 2013). These studies revealed an explanation for some porphyria-promoting drug side effects as well as the potential for environmental contributions to confound genotype/phenotype correlations. These demonstrations indicate that small molecule modulation of equilibria of morpheein forms is a viable approach to drug discovery. Nevertheless, a computational approach to identifying such modulators requires molecular resolution models and/or structures of alternate assemblies; these are not yet available for most putative morpheeins.

## Conclusion and Future Outlook

Proteins that can come apart, change shape, and reassemble differently with functional consequences provide expanded opportunities for understanding disease and designing therapeutics. This fifth level of protein structure provides another example where the one sequence/one structure/one function rule fails to provide the correct framework for data interpretation. Biophysical techniques that are becoming more widely available, (e.g., SEC-MALS, SEC-SAXS, cryo-EM, light scattering, to name just a few) are revealing the shape changing behavior of many disease-associated multimeric proteins. Other forms of microscopy are revealing changes in protein locations within cells. In some instances, these are associated with protein filamentation. All of these observations suggest the potential for harnessing the fifth level of protein structure for therapeutic advantage. As described, a few notable examples include ribonucleotide reductase (Thomas et al., 2019; Long et al., 2020), HIV integrase (Andrake and Skalka, 2015; Gupta et al., 2016), and Ebola VP40 protein (Bornholdt et al., 2013; Del Vecchio et al., 2018).

## Author Contributions

The author confirms being the sole contributor of this work and has approved it for publication.

## Conflict of Interest

The authors declare that the research was conducted in the absence of any commercial or financial relationships that could be construed as a potential conflict of interest.

## Footnotes

1 http://en.wikipedia.org/wiki/Morpheein
